# Metastatic Colorectal Cancer Patient with Microsatellite Stability and Germline BRAC2 Mutation Shows a Complete Response to Olaparib in Combination with a PD-1 Inhibitor and Bevacizumab: A Case Report and Review of the Literature

**DOI:** 10.3390/life13051183

**Published:** 2023-05-15

**Authors:** Minghan Song, Xianrong Zeng, Qian Wu, Jie Huang, Jiayi Dong, Lijuan Shao, Zihao Sun, Yiguang Lin, Size Chen

**Affiliations:** 1 Department of Immuno-Oncology, The First Affiliated Hospital of Guangdong Pharmaceutical University, Guangzhou 510080, China; smh_1997@163.com (M.S.);; 2 Guangdong Provincial Engineering Research Center for Esophageal Cancer Precision Therapy, Guangdong Pharmaceutical University, Guangzhou 510080, China; 3 Key Laboratory of Cancer Immunotherapy of Guangdong Higher Education Institutes, Guangdong Pharmaceutical University, Guangzhou 510080, China; 4 Department of Oncology, The Fifth Affiliated Hospital of Jinan University, Heyuan 517000, China; 5 Guangzhou Anjie Biomedical Technology Co., Ltd., Guangzhou 510530, China

**Keywords:** colorectal cancer, immunotherapy, PARP inhibitor, BRCA2 mutation, microsatellite stability, case report

## Abstract

Metastatic colorectal cancer (mCRC) has a poor prognosis. Combining chemotherapy with targeted therapy constitutes a basic form of mCRC treatment. Immune checkpoint inhibitors have been recommended for microsatellite instability mCRC, while most patients harboring microsatellite stability (MSS) or proficient mismatch repair (pMMR) are less responsive to immunotherapy. Combinational targeted therapy, including poly-ADP ribose polymerase (PARP) inhibitors, has been considered a promising way to reverse immunotherapy resistance; however, there is no clear and consistent conclusions can be drawn from the current research. Here, we report the case of a 59-year-old woman diagnosed with stage IVB MSS mCRC who received three courses of capecitabine/oxaliplatin chemotherapy combined with bevacizumab as a first-line treatment, resulting in an overall evaluation of stable disease (−25.7%). However, the occurrence of adverse events of intolerable grade 3 diarrhea and vomiting forced the cessation of this therapy. A germline *BRCA2* mutation was found by next-generation sequencing, and the patient further received a combination of olaparib, tislelizumab, and bevacizumab. This treatment regime resulted in a complete metabolic response and a partial response (−50.9%) after 3 months of treatment. Mild asymptomatic interstitial pneumonia and manageable hematologic toxicity were two adverse events associated with this combination therapy. This study provides new insights into the combination of PARP inhibitors and immunotherapy for MSS mCRC patients carrying germline *BRCA2* mutations.

## 1. Introduction

Colorectal cancer (CRC) maintains its position as the third most common cancer and the second leading cause of cancer death worldwide [1]. Almost 20% of newly diagnosed CRC patients have metastatic lesions, and another 25% will develop delayed metastases [2]. Although there have been significant improvements in the management of metastatic colorectal cancer (mCRC), including the addition of the vascular endothelial growth factor (VEGF) inhibitor bevacizumab or the epidermal growth factor receptor (EGFR) inhibitor cetuximab to the backbone of first-line chemotherapy, 5-year survival in patients with distant metastatic lesions is only 14%; therefore, outcomes for mCRC patients need significant improvement [3,4].

Almost 95% of mCRC patients harboring microsatellite stability (MSS) or proficient mismatch repair (pMMR) have a poor prognosis due to nonresponse to immune checkpoint inhibitors (ICIs), and combining molecular targeting treatment is the vital direction to reverse resistance to immunotherapy [5,6,7].

Olaparib is the first-generation poly-ADP ribose polymerase (PARP) inhibitor approved by the US Food and Drug Administration (FDA) to treat homologous recombination deficiency (HRD) in ovarian, breast, and pancreatic cancers [8,9,10]. A growing body of research has indicated the effectiveness of olaparib in CRC treatment [11,12]. Bevacizumab is an important targeted drug for first-line treatment and the maintenance treatment of CRC [13]. A recent study indicated that bevacizumab can improve the sensitivity of tumor cells to olaparib through hypoxia-induced DNA repair defects [14]. Preclinical studies and clinical trials also revealed that PARP inhibitors and ICIs have synergistic effects [15,16,17]. Relevant studies are ongoing to combine PARP inhibitors with ICI and antiangiogenic drugs [18].

Here, we present a case of a 59-year-old female patient presenting with MSS and *BRCA2*-mutated mCRC who was successfully treated with the combination of olaparib, tirelizumab, and bevacizumab. This case report demonstrates the potential synergy of combined immunotherapy in the treatment of CRC.

## 2. Case Description

**Figure 1:**
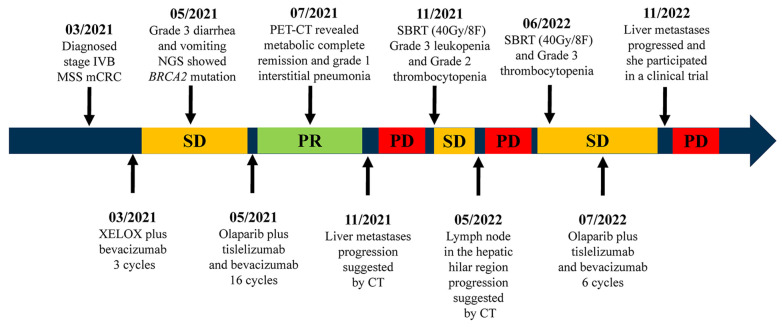
The timeline of the case shows the diagnosis, treatment, progression, and prognosis of the patient, and key adverse reactions of the treatment.

**Figure 2:**
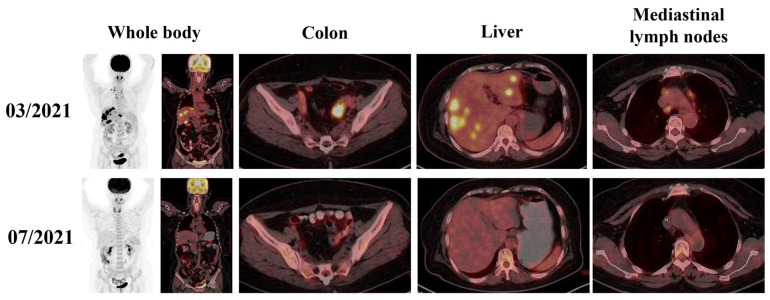
PET/CT scans show the changes in metabolic activity of the whole body, colon, and liver metastases of the patient. In the baseline time (March 2021), PET/CT scans revealed a colorectal tumor (SUVmax, 16.4; SUVave, 10.2), multiple liver metastases (SUVmax, 23.9; SUVave, 13.9), and multiple lymph node metastases (SUVmax, 7.5; SUVave, 4.5). After first-line chemotherapy and three courses of combination treatment, a complete metabolic response was achieved.

**Figure 3:**
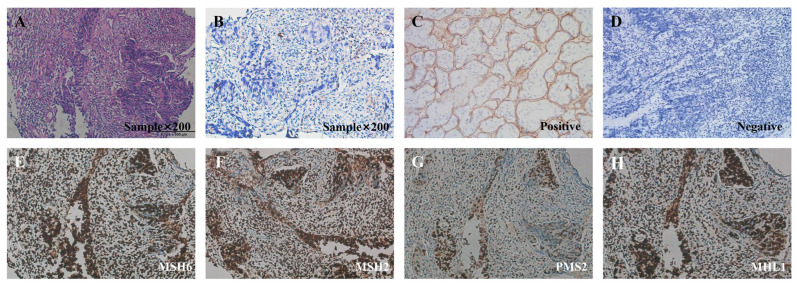
Immunohistochemical staining for detecting PD-L1 and MMR. (**A**) H&E sample under × 200 magnification showed infiltrative growth of colorectal adenocarcinoma cells. (**B**) PD-L1 expression was negative (TPS < 1%). Staining was performed using a Dako 22C3 assay kit following the manufacturer’s instructions. (**C**) Positive and (**D**) negative controls were under × 200 magnification. The expressions of all the MMR proteins were positive, including (**E**) MSH6(+), (**F**) MSH2(+), (**G**) PMS2(+), and (**H**) MHL1(+) under × 200 magnification.

In March 2021, a 59-year-old Asian female patient with latent syphilis was diagnosed with stage IVB CRC with multiple organ metastases. The patient presented with persistent abdominal pain and diarrhea, which had been ongoing over the past few months (the timeline covering diagnosis, treatment, progression, and prognosis of this patient is briefly shown in Figure 1). Family medical history revealed that the patient’s grandmother died from lung cancer. The physical examination was normal. Upon conducting laboratory tests, the tumor markers CA724, CEA, and CA19-9 were as high as 12.7 U/mL, 38.8 ng/mL, and 463.9 U/mL, respectively (Supplementary Figure S1A). A colon tumor (41 mm × 38 mm) and multiple distinct metastatic sites in the liver (S5, 39 mm × 42 mm; S8, 23 mm × 23 mm) and lymph node were identified via 18F-fluorodeoxyglucose positron emission tomography/computed tomography (18F-FDG-PET/CT) scans (Figure 2). Histology revealed a colorectal adenocarcinoma and immunohistochemistry (IHC) revealed pMMR (MSH6, MSH2, PMS2, and MHL1) positive expression and negative PD-L1 expression (TPS < 1%) (Figure 3).

**Figure 4:**
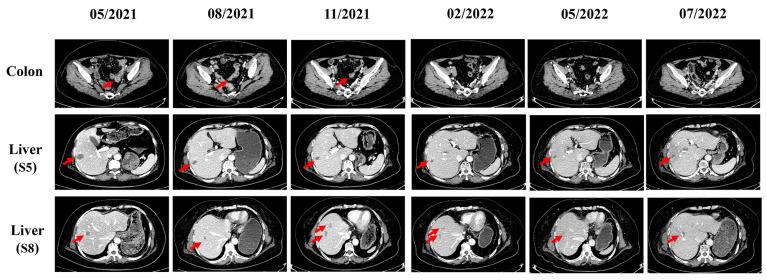
CT scans show the response of second-line and third-line therapies, including olaparib, a PD-1 Inhibitor, bevacizumab, and SBRT. The arrows refer to the location of the lesions (colon tumor and liver metastases). After combinational treatment involving olaparib with a PD-1 Inhibitor and bevacizumab, shrunken tumor was observed in August 2021 (colon tumor: length, 21 mm; liver metastases: S5, 20 mm × 15 mm; S8, 10 mm × 11 mm). In November 2021, the number of liver metastases increased. After combining SBRT, liver metastases gradually shrunk (S5, 17 mm × 14 mm; S8, 14 mm × 13 mm in July 2022).

The patient received three courses of capecitabine/oxaliplatin chemotherapy combined with bevacizumab (XELOX-bevacizumab) as a first-line treatment on 13 March 2021. A CT reexamination revealed an evaluation of stable disease (SD) (−25.7%) (RECIST ver 1.1) after two-course treatment. However, in May 2021, while taking capecitabine orally at home, the patient experienced grade 3 diarrhea and vomiting which had a dramatic impact on quality of life. Due to prolonged diarrhea and vomiting, the patient developed severe hypokalemia (serum potassium 2.81 mmol/L) and hypoproteinemia (serum albumin 24.1 g/L). This forced us to discontinue the capecitabine treatment. The possibility of colonic obstruction or tumor progression was ruled out by abdominal X-ray and CT scans (Figure 4 and Figure S2A). The patient received a series of supportive and symptomatic treatments, including antidiarrhea, antiemetics, antacids, analgesics, potassium vitamin supplementation, amino acids, and albumin. Additionally, the patient refused any intravenous chemotherapeutic drugs after experiencing severe adverse reactions. To explore potential targeted treatment, next-generation sequencing was performed in the patient’s peripheral blood. The patient was found to carry a germline mutation in *BRCA2* (c.4354del; pQ1452Rfs*11), low microsatellite instability (MSI) score, low tumor mutation burden (TMB, 1.68 Muts/Mb) and KRAS, NRAS, BRAF, and EGFR-wild types (Supplementary Figure S2B–D).

Previous studies demonstrated the refractory nature of multiple organ metastasis CRC [2], the limitations of PARP inhibitor monotherapy in CRC [11], and the potential synergistic effect between PARP inhibitors and ICIs [17]. Therefore, after a multidisciplinary team meeting and upon obtaining informed consent from the patient and her family, on 16 May 2021, combination therapy, consisting of a PARP inhibitor (olaparib, 600 mg daily) and a PD-1 inhibitor (tirelizumab 200 mg q3w), commenced. Bevacizumab was used as a maintenance therapy. On 13 July 2021, after three courses of this combination treatment, a follow-up PET/CT revealed a complete metabolic response, and tumor markers decreased into the normal range (Figure 2 and Figure S1A). In August 2021, follow-up CT scans showed that the colon tumor (length, 21 mm) and liver metastases (S5, 20 mm × 15 mm; S8, 10 mm × 11 mm) had shrunk further and a partial response (−50.9%) was assessed (Figure 4). During this time, adverse effects of mild anemia and grade 1 interstitial pneumonia occurred (Supplementary Figures S1B and S3). This combination therapy resulted in 5.4 months of progression-free survival (PFS) and, importantly, a significantly longer survival time of greater than 26 months compared to the median OS of 10.3–15.4 months for this subgroup of patients [19,20,21,22].

However, CT scans conducted in November 2021 revealed the recurrence of liver metastases (Figure 4). Therefore, combined stereotactic body radiotherapy (SBRT) (40Gy/8F) with systemic therapy was applied according to previous studies [23,24,25]. During this period, olaparib was reduced to 450 mg daily due to grade 3 leukopenia and grade 2 thrombocytopenia, while G-CSF support and eltrombopag were used to alleviate hematological toxicity. On 30 May 2022, PET/CT showed a new hypermetabolic lymph node in the hepatic hilar region, and the patient received a further dose of SBRT (40Gy/8F). Grade 3 thrombocytopenia occurred during combined radiotherapy, and olaparib was reduced to 300 mg daily in June 2022. After 5 months of stable disease, liver metastases reprogressed upon conducting CT scans in November 2022, and the patient participated in a clinical study of AK104/cadonilimab plus bevacizumab and capecitabine in relapsed and refractory mCRC. At the time of the last follow-up call in April 2023, the patient was still alive.

## 3. Discussion

This case reports an MSS mCRC patient with a germline *BRAC2* mutation who responded well to a combination therapy composed of olaparib, tirelizumab, and bevacizumab, indicating the importance and effectiveness of this combined immunotherapy in MSS mCRC.

MSS mCRC, accounting for more than 95% of mCRC, is considered as a specific kind of molecular subtype that was refractory to immune monotherapy due to the “cold tumor microenvironment”, and how to transform the “cold tumor microenvironment” into a “hot tumor microenvironment” is the main research direction at present [5,7,26,27]. Some studies have focused on the combination of ICIs with molecular targeted therapy [5]. Unfortunately, the phase II BACCI trial (NCT02873195) showed few clinical benefits of adding atezolizumab to capecitabine and bevacizumab therapy in patients with refractory mCRC, showing the preliminary failure to combine immunotherapy with bevacizumab in mCRC [22]. Although a small range of studies reported a synergistic effect of combining immunotherapy with EGFR inhibitors or tyrosine kinase inhibitors, further proof is still required in large prospective clinical trials [28,29]. Therefore, vast improvement and innovation is needed in combined immunotherapy for MSS mCRC.

Recent studies have shown that PARP inhibitors, as special enzyme inhibitors targeting DNA damage repair (DDR), can enhance the efficacy of immunotherapy in CRC through a variety of pathways [16,17,30]. PARP1/2 enzyme is the core DNA damage sensor and signal transducer in DDR, which is very important in the process of single-strand repair. PARP inhibitors can weaken the activity of the PARP1/2 enzyme, interfere with the normal repair process of broken DNA single strand, and have lethal effects with HRD synthesis, resulting in a cumulative increase in DNA double-strand breaks in tumor cells that cannot be repaired or unsustainable repair, thus mediating tumor cell death [17]. On the one hand, PARP inhibitors can mediate the accumulation of neoantigens through extensive DNA damage and directly increase the mutational burden in tumors, thereby driving the response to ICIs [31,32]. On the other hand, unrepaired double-strand DNA activates the stimulator of the interferon genes signaling pathway through the cytosolic DNA sensor cyclic GMP-AMP synthase to up-regulate type I interferon and PD-L1 expression and promote profound anti-tumor immune responsiveness [33,34]. In clinical studies, PARP inhibitors combined with immunotherapy have achieved considerable efficacy in ovarian and breast cancer. The results of a phase II trial (NCT02484404) of PARP inhibitors combined with ICIs in relapsed ovarian cancer showed that the overall response rate (ORR) was 14% (5/35) and the disease control rate (DCR) was 71% (25/35), indicating significant clinical activity of combining duvalumab with Olaparib in relapsed ovarian cancer regardless of *BRCA* status [35]. In a phase I trial (NCT02660034) of pamiaparib combined with tislelizumab in patients with advanced solid tumors, ORR was 20% (10/49), including two patients with complete response [36].

Although PARP inhibitors have been widely used in HRD breast and ovarian cancer with good clinical efficacy, there is no consistent research conclusion on the application of PARP inhibitors in CRC. Previous studies have shown that CRC is a potentially high HRD phenotype, with DDR gene mutation rates of 21% [37], HRD gene mutation rates of 15% [38], and BRCA1/2 mutation rates ranging from 1% to 8% [39,40]. In a recent large retrospective cohort study of 9321 CRC patients, the HRD subgroup accounted for approximately 13.6%, and after stratification by MMR status, the researchers found that more than 75% of microsatellite instability-high patients (MSI-H) and 9.5% of MSS patients had at least one HRD gene mutation [41]. These results indicate the great potential of PARP inhibitors in the treatment of CRC. PARP inhibitors were initially investigated as sensitizers of DNA damage in chemotherapeutic drugs for CRC treatment [42]. The first phase II trial (NCT00912743) involving 33 CRC patients who received olaparib after the failure of standard therapy showed that single-agent olaparib had few anti-tumor activities in homologous recombination-proficient (HRP) CRC patients [11]. IND 187 trial (NCT00535353) explored the combination of PARP inhibitors with irinotecan in CRC and concluded that both doses of olaparib and irinotecan should be reduced to prevent excessive toxicity and achieve efficacy [12]. Ghiringhelli et al. [43] and Papageorgiou et al. [44] also reported effective cases of combining PARP inhibitors with or without irinotecan in the treatment of patients with *ATM*-mutated or *CHECK2*-mutated refractory mCRC, and the best evaluation of efficacy was stable disease while PFS was prolonged by approximately 4 months. Subsequently, a preclinical study found that bevacizumab promoted the response to PARP inhibitors by creating a hypoxic tumor microenvironment to decrease *BRCA1/2* expression, thereby impairing HR repair [14,45]. However, the phase II trial (NCT02305758) combining veliparib with FOLFIRI and bevacizumab in CRC showed that the addition of veliparib to standard treatment did not prolong PFS or overall survival (OS) [46]. With the rise of immunotherapy, the combination of PARP inhibitors and ICIs has been explored in CRC. In preclinical research, Ghonim et al. found a synergistic effect of PARP inhibitor-based metrometric therapy with ICIs, with 78% (7/9) of MSI-H mice achieving complete tumor regression, and further studies found that olaparib enhanced the efficacy of ICIs by reducing the immunosuppressive function of myeloid-derived suppressor cells (MDSCs) [15]. Seyedin et al. found that the addition of veraparib to a combination of radiotherapy and immunotherapy significantly delayed tumor growth in mice [47]. A recent study showed that the concurrent administration of PARP inhibitors and ICIs in an immunocompetent *BRCA*-mutated CRC mouse model up-regulated type I and type II interferon pathways and increased CD4+T cells and CD8+T cells infiltration in the tumor microenvironment [48]. Currently, three phase I/II trials (NCT05201612, NCT02484404, and NCT03851614) are researching the potential synergism of combining PARP inhibitors and ICIs with or without bevacizumab in CRC, and the final results are pending.

In this case, report, the patient had many features of non-response to immunotherapy, including negative PD-L1 expression, low TMB, and MSS. However, after detection of the *BRCA2* mutation and receiving combined immunotherapy, the patient achieved a complete metabolic response, indicating the potent combined antitumor activity of PARP inhibitors and immunotherapy in MSS mCRC patients with *BRCA2* mutation. Although the patient achieved complete remission of systemic metabolic activity, five months after combined immunotherapy, liver metastasis progressed. Since the primary colon tumor in this patient was still in complete remission at the time of liver metastasis progression, we hold the opinion that the patient’s resistance to combination therapy could be related to the liver tumor’s own high malignancy and resistance to immunotherapy. As a result of the patient’s unwillingness to undergo another biopsy, we did not perform further tumor microenvironment detection and gene sequencing of the progressive tumor tissue to identify the reason for the patient’s resistance to the combined immunotherapy.

Potential adverse events still require attention based on the current results of synergistic anti-tumor efficacy in the combination of PARP inhibitors and ICIs in CRC. According to the results of multiple phase II trials of PARP inhibitors plus ICI in ovarian, breast, and prostate cancer, the most common adverse events were nausea and hypocytosis [16,49,50,51,52], and immune-related adverse effects with combination therapy were similar to those of patients with single agent immunotherapy [17]. For instance, the MEDIOLA phase II study (NCT02734004) investigating a combination treatment involving olaparib durvalumab in patients with BRCA-mutated metastatic cancers and among 40 included gastric cancer, grade 3 and grade 4 adverse events were 48% and 8%, and the most common adverse event of grade 3 or worse severity was anemia [52]. In this case report, mild hematologic toxicity and interstitial pneumonitis occurred during long-term oral olaparib in combination with immunotherapy and bevacizumab. In the face of aggravated hematologic toxicity after combining radiotherapy, the overall toxicity remained manageable with dose reduction and supportive care.

Overall, this case report affirms the synergistic effect of PARP inhibitors and immunotherapy in MSS mCRC patients with *BRCA2* mutation for the first time. Clearly, the combination therapy provided significant survival benefits while the overall toxicity was manageable, and the addition of PARP inhibitors did not increase new adverse reactions associated with ICIs. In agreement with our data, previous studies demonstrated that a combination of olaparib and durvalumab in the treatment of relapsed gastric cancer resulted in unexpectedly long survival [52]. The underlying mechanism of the combination therapy is not fully understood. It was believed to be associated with the decrease in immunosuppressive cells and the increase in CD4+ and CD8+ T cell infiltration in the tumor microenvironment, promoting the activation of an inflammatory immune microenvironment [48].

As shown in Figure 1 and Figure 4, disease progression occurred five months after combination therapy. We are not certain what caused this to occur at that time. One of the possible causes could be the development of drug resistance that makes the patient not responsive to immunotherapy and resistant to PARP inhibitors. Another possible cause may be related to the high degree of malignancy inherent in the liver tumor. We believe that this combination therapy is effective in the treatment of the primary focus of colon cancer, but liver metastases have a poor response to treatment. We were unable to perform further tumor microenvironment detection and gene sequencing of progressive tumor tissue to find out the underlying changes in the tumor tissues since the patient was unwilling to undergo another biopsy.

## 4. Conclusions

In summary, the present case suggests that a combination therapy combining targeted therapy (olaparib + bevacizumab) with immunotherapy (tislelizumab) is important in the treatment of MSS mCRC patients with germline *BRCA2* mutations. The treatment provides an effective treatment option for this subgroup and is well tolerated. This report also encourages further research into combining immunotherapy with targeted therapy in future large clinical trials to optimize management strategies for MSS mCRC patients.

## Data Availability

All data have been submitted in the manuscript and Supplementary Materials. If additional data are required, please contact the corresponding authors.

## Supplementary Materials

The following supporting information can be downloaded at: https://www.mdpi.com/article/10.3390/life13051183/s1, Figure S1: Variability of (A) tumor markers and (B) peripheral blood cell blood cells of this patient over time; Figure S2: The result of abdominal radiograph and NGS. (A) abdominal radiographs showed no signs of intestinal obstruction. (B) NGS showed BRCA2, TP53, and SH2B3 mutations and KRAS, NRAS, BRAF, and EGFR-wide types. NGS showed (C) low MSI score and (D) low TMB; Figure S3: Chest CT scans revealed an increase in cords and ground-glass opacities in both lower lungs field after combination therapy in August 2021 and diminished gradually.
