# Supplementary material for: Metastatic Colorectal Cancer Patient with Microsatellite Stability and Germline BRAC2 Mutation Shows a Complete Response to Olaparib in Combination with a PD-1 Inhibitor and Bevacizumab: A Case Report and Review of the Literature
Source: Life (Basel). 2023 May 15;13(5):1183. doi: 10.3390/life13051183 (PMC10222588; doi:10.3390/life13051183)
Supplement: Supplementary file 1 [file life-13-01183-s001.zip › life-2345820-supplementary.pdf]

Supplementary Materials

Supplementary Figures

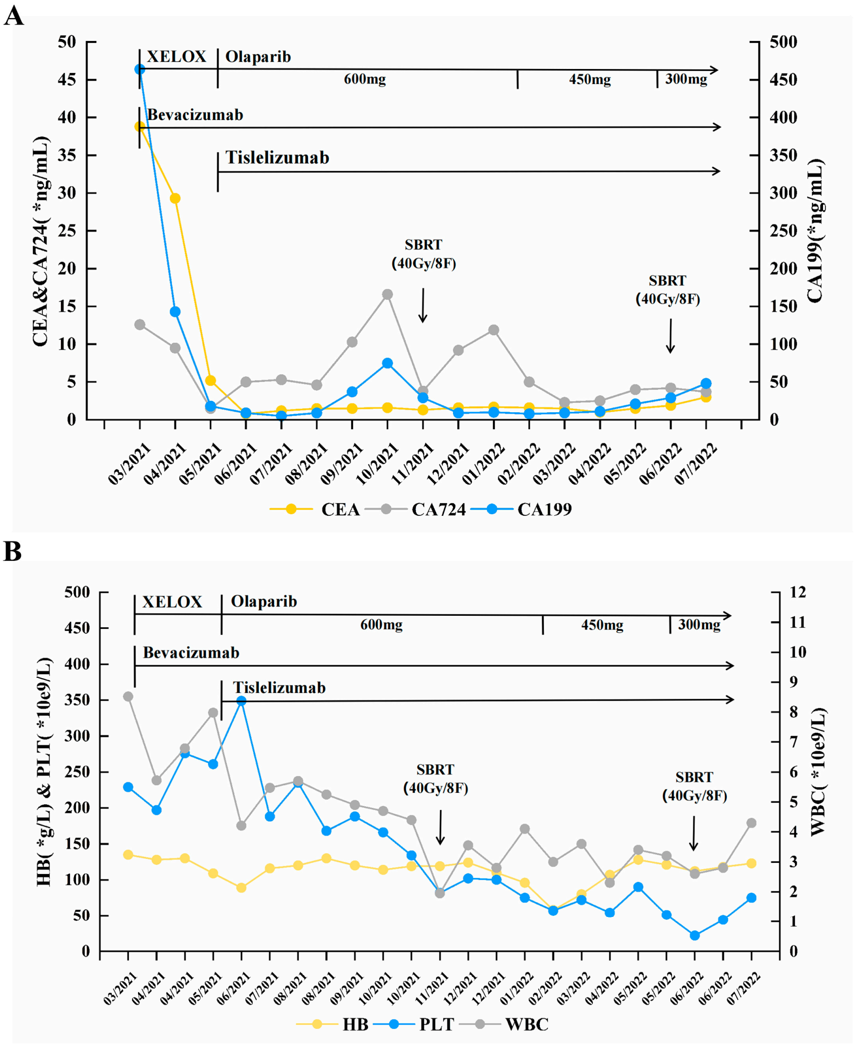

Supplementary Figure S1. Variability of (A) tumor markers and (B) peripheral blood cell blood cells of this patient over time.

A

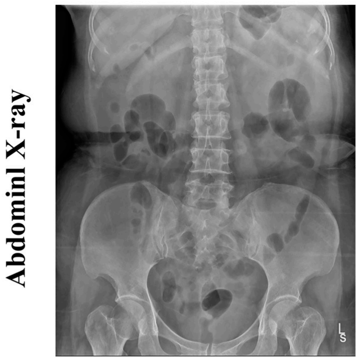

B

Gene	Source	Mutation style	Frequency
BRCA1	/	/	/
BRCA2	germline	p.Q1452Rfs*11	42.99%
TP53	somatic	p.H178P	5.22%
SH2B3	somatic	p.H158P	6.10%
BRAF	/	Wide	/
KRAS	/	Wide	/
NRAS	/	Wide	/
EGFR	/	Wide	/

C

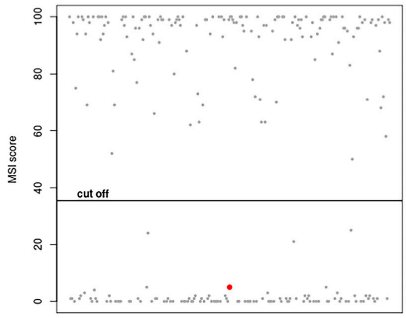

D

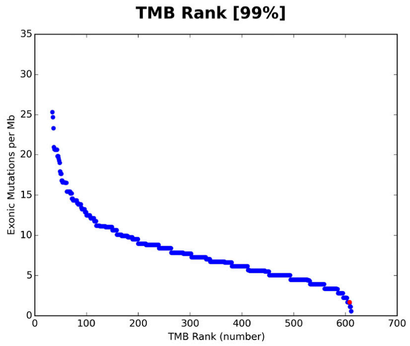

Supplementary Figure S2. The result of abdominal radiograph and NGS. (A) abdominal radiographs showed no signs of intestinal obstruction. (B) NGS showed BRCA2, TP53, and SH2B3 mutations and KRAS, NRAS, BRAF, and EGFR-wide types. NGS showed (C) low MSI score and (D) low TMB.

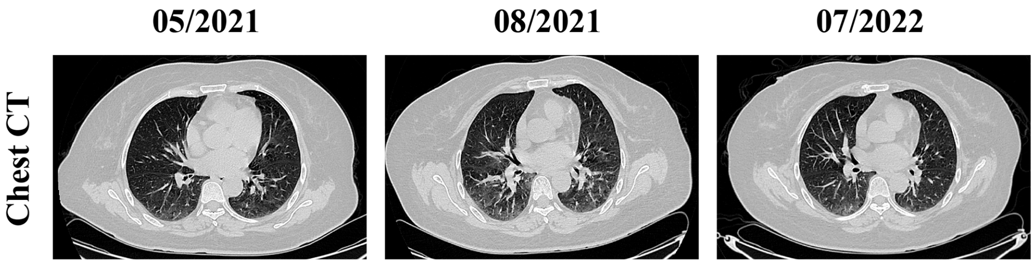

Supplementary Figure S3. Chest CT scans revealed an increase in cords and ground-glass opacities in both lower lungs field after combination therapy in August 2021 and diminished gradually.
